# Directed evolution for high functional production and stability of a challenging G protein-coupled receptor

**DOI:** 10.1038/s41598-021-87793-9

**Published:** 2021-04-21

**Authors:** Yann Waltenspühl, Jeliazko R. Jeliazkov, Lutz Kummer, Andreas Plückthun

**Affiliations:** Department of Biochemistry, University of Zürich, Winterthurerstrasse 190, 8057 Zurich, Switzerland

**Keywords:** G protein-coupled receptors, Biophysical chemistry, Expression systems, Protein design, Next-generation sequencing, Experimental evolution

## Abstract

Membrane proteins such as G protein-coupled receptors (GPCRs) carry out many fundamental biological functions, are involved in a large number of physiological responses, and are thus important drug targets. To allow detailed biophysical and structural studies, most of these important receptors have to be engineered to overcome their poor intrinsic stability and low expression levels. However, those GPCRs with especially poor properties cannot be successfully optimised even with the current technologies. Here, we present an engineering strategy, based on the combination of three previously developed directed evolution methods, to improve the properties of particularly challenging GPCRs. Application of this novel combination approach enabled the successful selection for improved and crystallisable variants of the human oxytocin receptor, a GPCR with particularly low intrinsic production levels. To analyse the selection results and, in particular, compare the mutations enriched in different hosts, we developed a Next-Generation Sequencing (NGS) strategy that combines long reads, covering the whole receptor, with exceptionally low error rates. This study thus gave insight into the evolution pressure on the same membrane protein in prokaryotes and eukaryotes. Our long-read NGS strategy provides a general methodology for the highly accurate analysis of libraries of point mutants during directed evolution.

## Introduction

G protein-coupled receptors (GPCRs) play crucial roles in human physiology^1,2^. This biological importance is further reflected in their therapeutic relevance. As such, GPCRs constitute the largest class of single drug targets, with an estimated 35% of marketed FDA approved drugs acting through these receptors^3,4^. In recent decades, substantial efforts have thus been invested to elucidate the structural rationales for aiding the development of therapeutics acting on GPCRs. For a long time, biophysical experiments, including structural studies, have been complicated by most of the receptors’ low natural abundance, low functional expression levels in recombinant hosts, and poor stability after removal from the membrane^5,6^.

To address these difficulties intrinsic to GPCRs, several methodologies have been developed, including advances in recombinant overexpression, purification strategies^7,8^, crystallization platforms^9,10^, and protein engineering^11–15^. This has increased the number of high-resolution structures, providing fundamental insights into the mechanistic understanding of at least some GPCRs^16,17^. To advance drug discovery programs and to obtain a more comprehensive understanding of GPCR dynamics, complementary approaches to the structural studies are also necessary, including techniques such as surface plasmon resonance (SPR)^18,19^ and nuclear magnetic resonance (NMR)^20^.

For such challenging experiments, GPCRs need to be sufficiently stable in detergent for a prolonged time period, and they need to be functionally expressible in a recombinant host suitable for isotope labelling. In addition, many receptors of great interest exhibit particularly low yields and/or low stability and have thus remained inaccessible to most biophysical and/or structural studies, despite the above-mentioned technological advances and the large interest in the field.

For the most challenging receptors, however, even the methods developed so far, when individually applied, have not been sufficient. In the present study, we have thus analysed the performance of these methods, and devised analysis tools to help understand why the serial use of different strategies of directed evolution is particularly successful.

We have previously developed several evolutionary technology platforms to overcome the biophysical limitations of challenging receptors^21–24^. These methods are all based on the powerful common principles of directed evolution: initially, the wild-type (wt) sequence of a protein is randomised, then, through application of a selection pressure, variants with increased functional expression^21,23,24^ or—directly—mutants with increased stability in detergent^22^ are enriched from a library. In these directed evolution experiments, the selection pressure is controlled by probing ligand binding with a fluorescently labelled ligand, thereby simultaneously ensuring functional folding of the receptor. The power of these directed evolution methods is underlined by the so enabled structural and functional studies of several reported GPCRs with improved the biophysical characteristics^21,22,24–28^.

The initially developed evolution methods require expression in *Escherichia coli* (*E. coli*). *E. coli*-based evolution^21^ for improving functional receptor surface expression is not applicable to all GPCRs—some GPCRs cannot be functionally expressed at any meaningful level, possibly due to the missing secretory quality control machinery of the eukaryotic cell and/or differences in membrane lipid composition. A second *E. coli*-based method, aimed at improving protein stability in detergent by cellular high-throughput encapsulation solubilization and screening (CHESS)^22^, i.e., by encapsulating *E. coli* cells and converting them into nanoscale dialysis tubes, is only accessible to receptors that can be functionally expressed in *E. coli*. These limitations were overcome with the development of a third selection method based on a eukaryotic host^24^: *Saccharomyces cerevisiae*-based receptor evolution (SaBRE) has opened the door to selection for challenging receptors that were not previously amenable to selection and evolution technologies in prokaryotic hosts. Nonetheless, SaBRE alone has also not been able to bring all receptors to the required level of stability.

We were interested in determining the structure of the human oxytocin receptor (OTR) and discovered that it is one of the most challenging targets for selection, as it expresses very poorly in almost all hosts—even in insect cells^29^. To solve this problem, we decided to combine our methods, and we hypothesized that the implementation of SaBRE makes previously inaccessible receptors subsequently amenable to CHESS.

Due to its biologically conserved role in organizing both sexual reproduction and social behavior^30^ the OTR is clinically targeted not only for preventing spontaneous premature labour, but also for the treatment of mental health disorders including Asperger’s syndrome and schizophrenia^31–34^. However, novel drug discovery programs targeting the OTR were so far complicated by the receptor’s challenging in vitro behaviour, and this would be greatly facilitated by identification of stable, well-expressing OTR mutants which could also be crystallised.

Here we present a strategy that successfully combines SaBRE and CHESS by bridging the two distinct evolution strategies with an additional series of selection steps to produce stable and highly expressing human OTR variants. First, we applied the SaBRE method, selecting for well-expressing OTR variants in a eukaryotic host (*S. cerevisiae*) from a randomised library of the wild-type OTR (wtOTR) gene. Then, we switched to *E. coli* as the selection host. We pursued several rounds of selection for functional expression and stability in *E. coli* prior to applying the CHESS approach, where variants are selected for stability in detergent. Crucially, CHESS would not have been possible with a direct transition from SaBRE-selection in *E. coli* for functional expression is an essential intermediate step*.*

To investigate exactly why these additional steps are necessary, we wished to trace the directed evolution across the eukaryotic and prokaryotic host. We wanted to use next-generation sequencing (NGS), but to do so we had to overcome the challenge of requiring extremely long reads (the whole ORF of the GPCR) with extremely high accuracy. For this purpose, we developed a high-throughput sequencing pipeline based on single-molecule real-time (SMRT) sequencing with careful intrinsic control of technical sequencing read errors. The long reads generated by this particular NGS approach allowed sequencing of full-length OTR variants, and therefore information of mutational linkage was maintained. Overall, we generated more than 55,000 unique sequences, which allowed the identification of critical mutations in the OTR gene important for the overall selection success.

## Results

### Evolution of well-expressing OTR variants

It is not uncommon for GPCRs to have poor biophysical properties. One such GPCR is the human OTR, which has very low intrinsic stability and especially low functional expression levels^29,35^. When wild-type OTR (wtOTR) is overexpressed in *E. coli* or *S. cerevisiae*, no surface expression is detected in flow cytometry experiments (data not shown). In fact, wtOTR even appears to be toxic to *E. coli* cells, even when expressed under optimised conditions, i.e., low-copy number plasmid, low temperatures, and with N- and C-terminal fusion proteins to help translocation of the overexpressed receptor to the inner membrane. The toxicity of OTR overexpression to *E. coli* might be related to the lack of cholesterol in *E. coli* membrane, which is equally important for OTR stability and integrity^36,37^. The poor biophysical behaviour of the wtOTR as a starting structure and its biological importance make it an ideal target to employ previously developed engineering strategies for increasing expression and stability, such as CHESS^22^.

**Figure 1:**
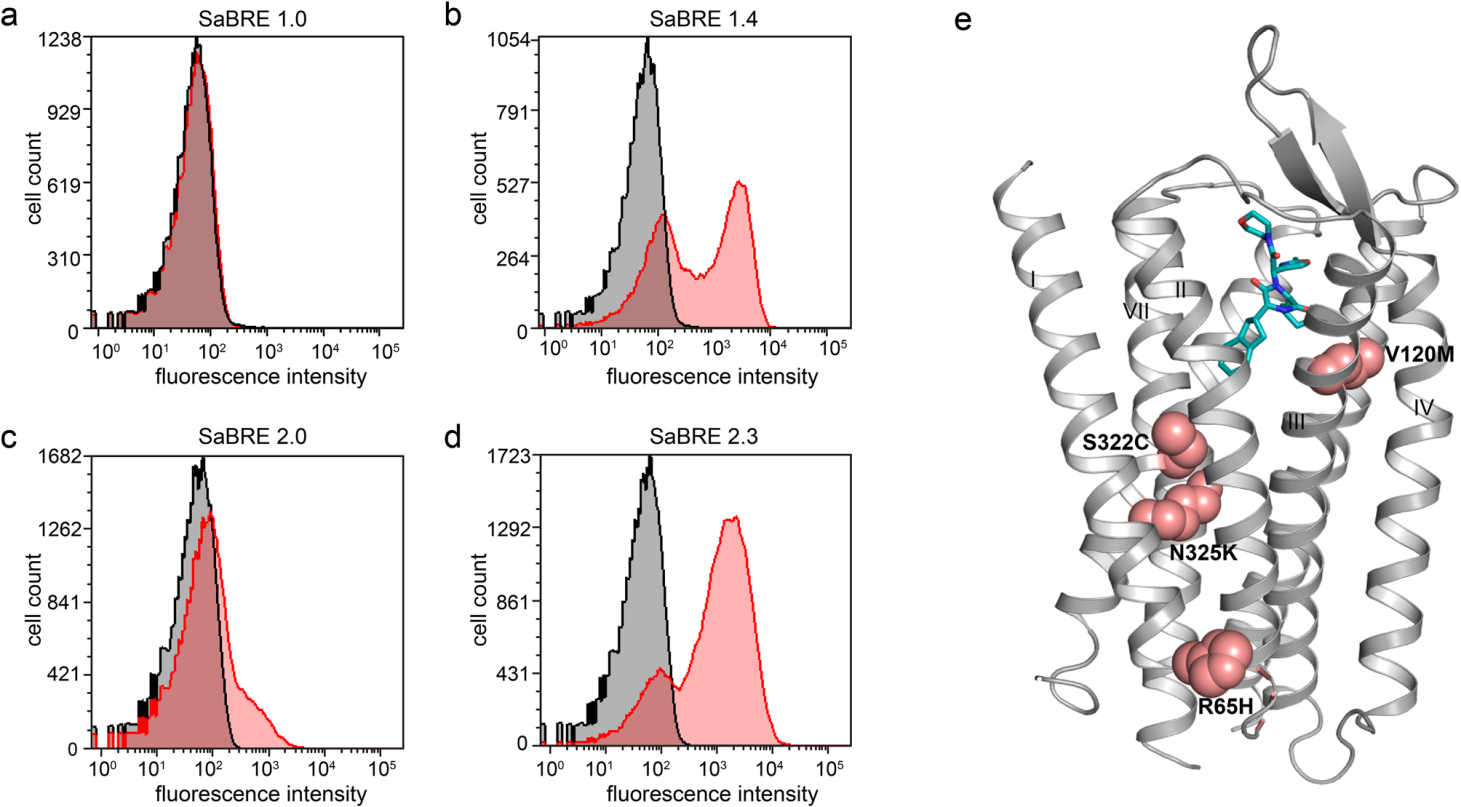
Identification of well-expressing OTR variants with SaBRE. (**a**,**b**) Histogram plots of fluorescent ligand binding data measured by flow cytometry. The total signal (red curves) and nonspecific signal (in the presence of unlabelled ligand, black curves) are shown for the starting pool SaBRE 1.0 (**a**) and the final pool SaBRE 1.4 (**b**) of the first round of SaBRE. (**c**,**d**) Histogram plots of fluorescent ligand binding flow cytometry data with total signal (red curves) and nonspecific signal (black curves) are shown for the starting pool SaBRE 2.0 (**c**) and the final pool SaBRE 2.3 (**d**) of the second round of SaBRE. (**e**) Mutations (pink spheres) of the single clone, OT-y01, identified in pool SaBRE 1.4, mapped on the OTR structure (grey, PDB ID: 6TPK) in complex with retosiban (turquois).

However, to perform CHESS with the OTR, it has first to be expressible in *E. coli* at least at some modest level, which is not the case. Thus, to obtain receptor variants that can be expressed in *E. coli*, we chose a successive selection strategy starting with SaBRE, using yeast as a eukaryotic expression host, before switching to a prokaryotic expression system. For selection with SaBRE, we randomised the wtOTR gene with an error-prone polymerase chain reaction (epPCR). *S. cerevisiae* was subsequently transformed with the created DNA library (SaBRE 1.0). The library was expressed, and surface expression was probed with the fluorescently labelled peptide antagonist PVA^38^ (HiLyte Fluor 647-Lys^8^ PVA). However, no specific signal was observed for the naïve library, even at several different ligand concentrations tested, well above the dissociation constant of the ligand (Fig. 1a, Supplementary Fig. S1). On the contrary, we observed an increasing nonspecific signal at increasing fluorescent ligand concentrations, indicating that high concentrations of labelled ligand complicate the selection process.

Therefore, the highest concentration of fluorescent ligand where single specific events were still observed in flow cytometry was used to probe receptor expression levels during selection. In the first round of SaBRE (Supplementary Fig. S2a), a very stringent gating strategy was pursued to maximise selection of specific binding events. After three consecutive sorting steps comprising re-cultivating sorted yeast cells, re-expression of OTR variants, and selection with fluorescence-activated cell sorting (FACS), a pool was enriched (SaBRE 1.4) that showed significantly increased surface expression (Fig. 1b). From this pool, plasmid DNA was isolated and multiple clones were sequenced, but only one OTR variant (termed OT-y01) was identified multiple times. This single enriched clone contained five amino acid point mutations (A19^N-term^T, R65^1.58^H, V120^3.33^M, S322^7.46^C and N325^7.49^K; Ballesteros and Weinstein numbering^39^ denoted as superscript). With the exception of A19T, all mutations are located within transmembrane (TM) helix interfaces and most likely contribute to improved helix packing (Fig. 1e).

To generate a more diverse starting point for selection in *E. coli* or CHESS, an additional round of SaBRE was conducted (Supplementary Fig. S2). OT-y01 was randomised once again by epPCR and *S.* *cerevisiae* was transformed with the created library (SaBRE 2.0). This library already showed an increased expression level when probed in flow cytometry compared to the initial library SaBRE 1.0 (Fig. 1a,c). In total, four subsequent steps of enrichment were carried out after the second randomization (Fig. 1d, Supplementary Fig. S1a). The plasmid DNA from the last two rounds of SaBRE (SaBRE 2.3 and SaBRE 2.4) were isolated, and 48 clones from each pool were sequenced and analysed. Sequencing determined that the SaBRE 2.3 pool has greater diversity (43 individual variants, with the most enriched clone appearing twice) compared to the SaBRE 2.4 pool (30 individual clones, with the most enriched clone appearing nine times). The more diverse pool, SaBRE 2.3, was chosen as a starting point for switching the expression host from yeast to bacteria to allow the broadest possible sampling of variants.

**Figure 2:**
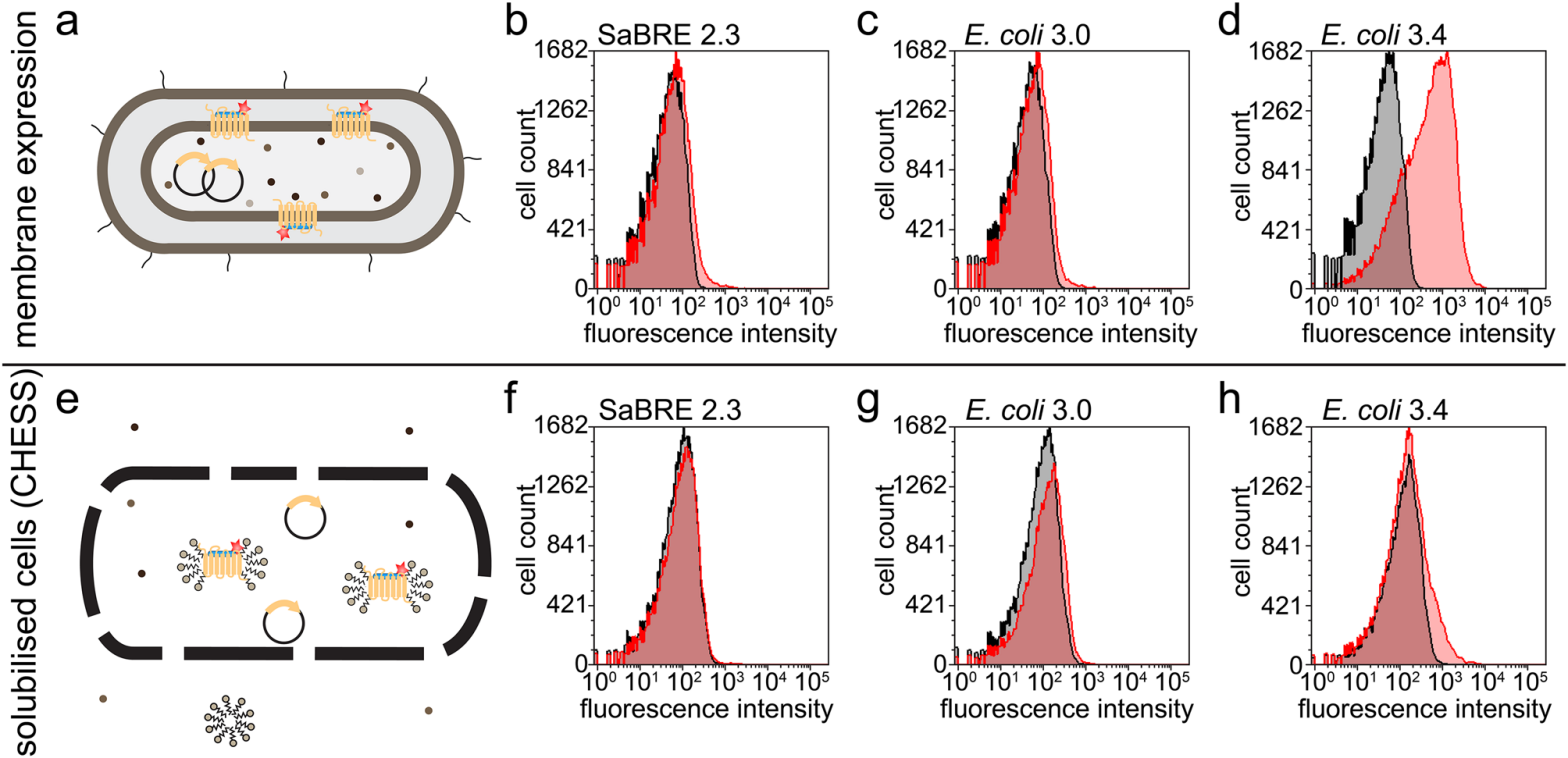
Selection for high functional expression and detergent stability in *E. coli.* (**a**) Schematic representation of the probing with fluorescently labelled (red star) ligand (blue rod) for well-expressing OTR variants (yellow rods) in the membrane of intact *E. coli* cells. (**b**,**d**) Histogram plots of flow cytometry detecting binding of fluorescent ligand on intact *E. coli* cells. Total signal (red curves) and nonspecific signal (black curves) are shown for the pools SaBRE 2.3 (**b**) *E. coli* 3.0 (**c**) and *E. coli* 3.4 (**d**), expressed in *E. coli*. (**e**) Schematic representation of a polymer-encapsulated (black lines) *E. coli* cell, whose internal space is subsequently solubilised with detergent, as performed in CHESS, converting the cells essentially to nanoscopic dialysis bags. (**f**,**h**) Histogram plots of flow cytometry detecting binding of fluorescent ligand within polymer capsules. Total signal (red curves) and nonspecific signal (black curves) are shown for the pools SaBRE 2.3 (**f**) *E. coli* 3.0 (**g**) and *E. coli* 3.4 (**h**), after *E. coli* expression,encapsulation and solubilisation.

For this purpose, yeast-evolved OTR variants enriched in pool SaBRE 2.3 were isolated and subsequently *E. coli* was transformed with these constructs. However, only a poorly specific binding signal could be detected in *E. coli* for variants of the SaBRE 2.3 pool (Fig. 2a,b). While now functional expression was observed, the switch from *S. cerevisiae* to *E. coli* proved more difficult than expected. Unsurprisingly, no specific binding signal was consequently detected for variants enriched from the SaBRE 2.3 pool when probed in CHESS. In this method, the *E. coli* cells of the library are first encapsulated by a polymer layer, such that each cell is wrapped individually and subsequently solubilised in detergent (Fig. 2e,f). Thereby, each cell is converted into a nanoscopic dialysis bag.

,*E. coli*. OTR variants isolated from the SaBRE 2.3 pool were randomised a third time by epPCR and *E. coli* was transformed with this library (termed *E. coli* 3.0). The *E. coli*,2,*E. coli,* a notable increase in surface expression was observed (*E. coli*,2d). This pool also showed a clearly delineated binding signal shift when used in CHESS[22] (Fig. 2,*E. coli*,*E. coli*.

### Next-generation sequencing of selection pools

To analyse the mutations enriched in single OTR variants during selection, we initially determined the sequence of 48 clones (by Sanger sequencing) from pools SaBRE 2.3, SaBRE 2.4, *E. coli* 3.4 and *E. coli* 3.5. Surprisingly, all of the sequenced pools proved to be diverse. For example, the SaBRE 2.3 pool had a total of 43 unique sequences identified (38 singly occurring clones and 5 doubly occurring clones). High diversity precludes comprehensive analysis by Sanger sequencing, due to its small sampling size. To facilitate sequence analysis of the diverse pools enriched by evolution, we developed an NGS sequencing strategy for this purpose.

We required a sequencing strategy that fulfilled two main criteria: (1) the sequencing method must provide very accurate single reads to allow the identification of point mutations in a single variant, and (2) the sequencing platform must generate reads long enough to ensure sequencing of a single complete OTR gene in one stretch (read lengths > 1000 base pairs (bp)), thus allowing not only the identification of mutations but also capturing their co-occurrences. The method best matching these criteria was single-molecule real-time (SMRT) sequencing, offered by PacBio. In a SMRT sequencing experiment, single circularised gene variants are sequenced in individual wells, constituting zero mode waveguides (ZMW). A rolling circle amplification approach is used in which the complementary strand is synthesised by an immobilized polymerase, with fluorophore release upon nucleotide incorporation, achieving read lengths of up to 100,000 bp^40^. A single circularised OTR variant (1056 bp) present in a ZMW should result in a read with multiple passes (subreads) because of its comparably small length compared to the maximum read length of the rolling circle amplification approach. These subreads should later allow generation of accurate circular consensus sequencing (CCS) reads of the OTR variants present in the according ZMW.

**Figure 3:**
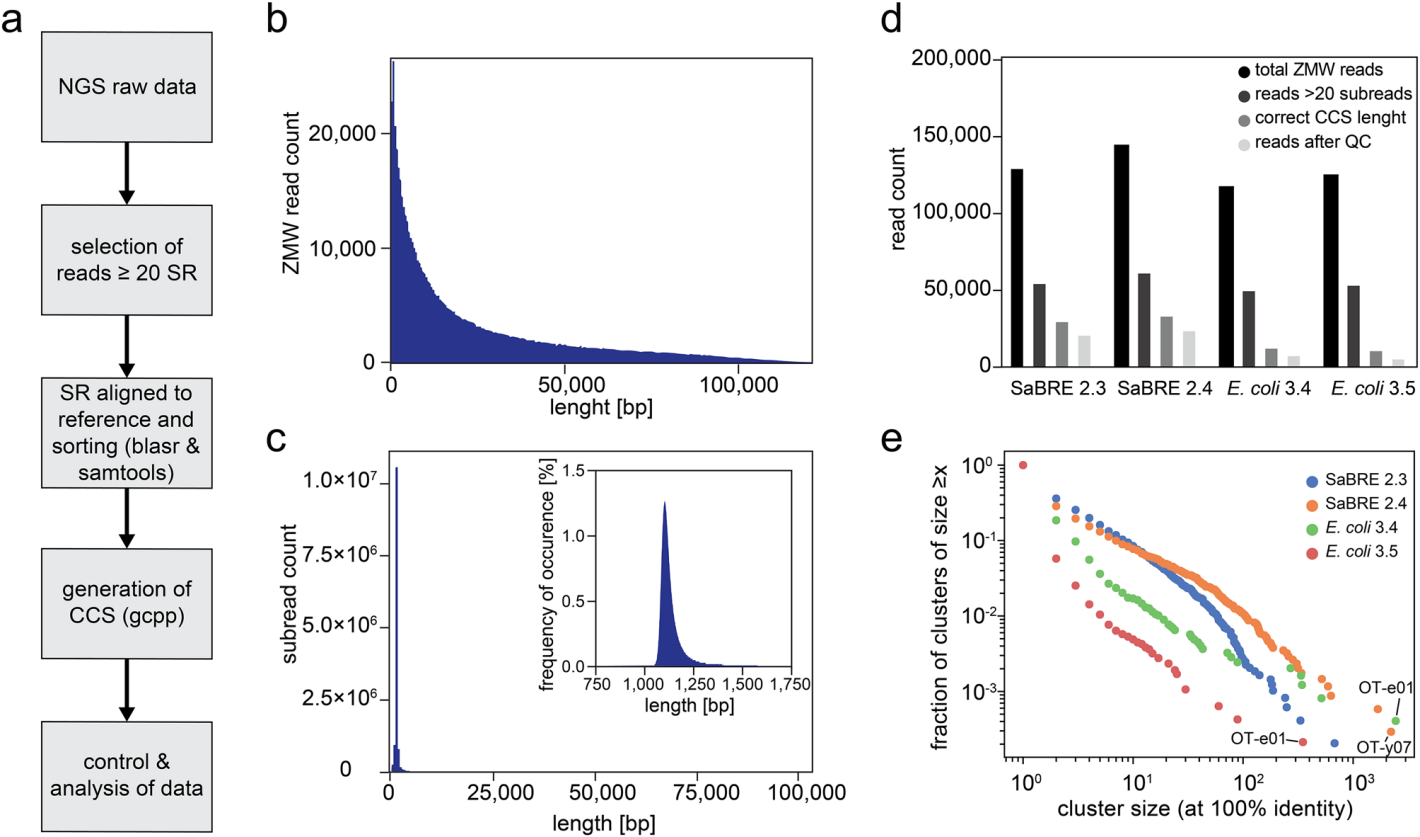
Single-molecule real-time sequencing of OTR selection pools. (**a**) NGS data processing work-flow. (**b**) Histogram representation of all reads and the respective read lengths generated during SMRT sequencing. (**c**) Histogram representation of all subreads and the respective subread lengths generated during SMRT sequencing. Blow-up of the main peak is depicted in the top right corner. (**d**) Diagram of read counts retained after the indicated processing steps (top right corner) for all four pools sequenced. (**e**) Scatter plot of the cluster sizes and the fraction of clusters observed within the indicated (top right corner) sequence pools*.* The cluster size represents the number of times a particular unique sequence is observed. The fraction of clusters is obtained by counting all clusters of size x divided by the total number of clusters.

The individual pools were simultaneously sequenced on a single SMRT cell using a PacBio Sequel instrument. A total of 624,145 ZMW reads were generated with a mean read length of 23,661 bp and a median subread length of 1,112 bp, resulting in a mean subread number of 20.6 (Fig. 3b,c). During data analysis we found, analogously to previous reports^41^, that individual subreads contained a high number of insertions and deletions (indels). These indels are not an expected by-product of our evolution strategy (and none have been found by Sanger sequencing from the same pool and other pools from other evolution projects), but rather a direct consequence of the sequencing approach. As incorrectly identified indels critically impact the quality of the sequence analysis by shifting the reading frame of the sequenced gene, we developed a novel analysis approach.

To overcome the low quality of the subreads and generate consensus sequences from single ZMW outputs with high accuracy, we implemented a processing strategy that included five steps (Fig. 3a). First, only reads with at least 20 subreads were considered. Second, the single subreads were aligned to the wtOTR gene sequence. The aligned subreads were then used to generate consensus sequences. Consensus sequences with incorrect read lengths were excluded from analysis, as we did not expect our evolution strategy to produce indels. In a final control step, consensus sequences were translated and the top 1% of sequences with the highest mutation rates, typically caused by a frameshift in the open reading frame, were excluded. Applying this stringent protocol on the raw sequencing data resulted in a retention rate of around 10% (Fig. 3d).

To test the validity and accuracy of the obtained sequences generated by this approach, we compared these NGS sequences with the sequences obtained from Sanger sequencing. For all four pools sequenced, we found almost identical mutation rates per gene and similar occurrences of single point mutations and amino acid changes on the protein level (Supplementary Fig. S3) when comparing the two sequencing methods. Furthermore, we found in the datasets of NGS sequences and Sanger sequences the same clones to be most enriched for both final pools (SaBRE 3.4 and *E. coli* 3.4) (Table S3). We thus concluded that the NGS sequences so obtained and processed had the same quality as the sequences obtained from Sanger sequencing.

Additional variations to the data processing pipeline, such as different software for alignment and consensus calling as well as the implementation of more stringent cut-offs, were tested without substantially improving the retention rate or accuracy (compared to Sanger sequencing).

Despite only 10% of all ZMW reads resulting in translatable consensus sequences of high accuracy, we still obtained 8000–20,000 sequences for each individual pool and thus a total of 58,050 sequences from one NGS run.

### In-depth analysis of enriched selection pools

**Figure 4:**
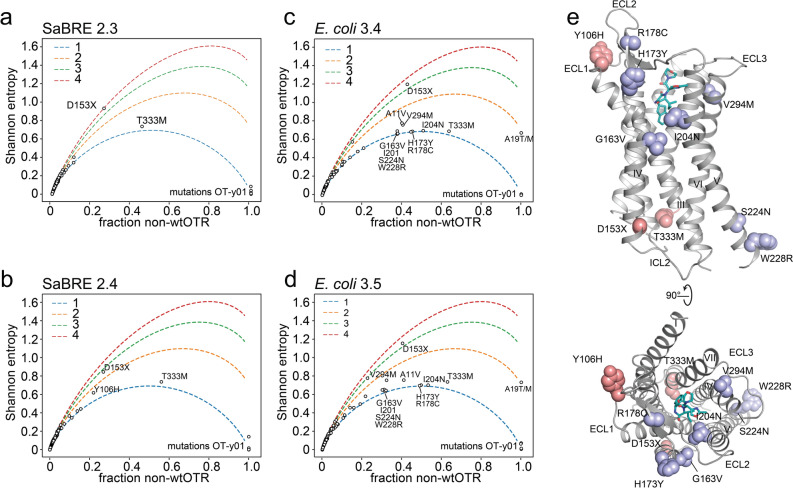
Observed mutational frequencies. (**a**–**d**) Shannon entropy calculated with Eq. (1) for every individual amino acid position (black circles) on the OTR, plotted against fraction of non-wtOTR amino acid occurrence. To help visualise the theoretical Shannon entropy for n = 1 (blue), n = 2 (orange), n = 3 (green) and n = 4 (red), non-wt amino acids observed are depicted as dashed lines. Individual positions being mutated with a higher frequency than the most enriched clone of a pool are indicated with a label, as they must have been independently selected several times. (**e**) Amino acids positions with a high non-wtOTR frequency are mapped on the OTR (grey, PDB ID: 6TPK) bound to retosiban (turquois). Mutations occurring at a high frequency after SaBRE are coloured pink, positions mutated at a high frequency after *E. coli*-based selection are coloured light purple.

Our NGS approach dissected a large number of variants suitable for an in-depth analysis of the selection outcome. We found a median mutational rate of 9 amino acid changes per OTR variant after SaBRE, and a median mutational rate of 12 amino acid changes per OTR variant in the *E. coli*-derived selection pools. Certain positions were enriched for mutations across the entire pool, independent of the specific OTR variant or of other mutations. Preponderance of single amino acid mutations at a particular position is likely due to their beneficial effect on the biophysical properties of the receptor. These mutations and positions were identified by calculating the frequency of the most dominant non-wt (f_non-wt_) amino acid occurring at every position on the OTR gene (Fig. 4, Supplementary Fig. S3).

Five amino acid positions are observed to all have single non-wt amino acid in 100% of analysed sequences. These mutations (A19^N-term^T, R65^1.58^H, V120^3.33^M, S322^7.46^C, and N325^7.49^K) stem from the single clone, OT-y01, used as the source for the second round of SaBRE and, while their occurrence is not surprising, their complete uniformity confirms the fidelity of NGS processing pipeline. In the selection outcome of SaBRE, the most frequently observed non-wt mutations are D153^4.42^N and T333^8.47^M, occurring at a frequency of 21% and 56%, respectively (Fig. 4a,b). Mutations in both these positions, D153^4.42^ and especially T333^8.47^, occur more frequently than the top three single clones (accounting for 19.5% of pool diversity in SaBRE 2.4, Supplementary Table S3), indicating their phenotypic selection in different sequence backgrounds. Both positions are located at the ends of transmembrane helices (TM 4 and TM 7, respectively) and both point into the receptor core (Fig. 4e). We hypothesise that these mutations might optimise helix–helix packing interactions. Our hypothesis is further strengthened by the observation these interactions are independent (i.e., the frequency of each observed amino acid pair is almost equal to the product of the individual frequencies, Supplementary Fig. S5) and this independence is not system-dependent—it is observed throughout the evolution process in *E. coli* and in *S. cerevisiae*.

In the final pool of the selection in *E. coli* (*E. coli* 3.4), position H173^4.62^Y (45%), R178^EL2^C (45%), and I204^5.42^N (51%) all occur at a higher frequency (Fig. 4c) than the most enriched clones of the corresponding pools (top three clones account for 41.1%, Supplementary Table S3), again underlining that they have been phenotypically selected in different backgrounds. I204 is pointing into the binding pocket of the receptor (Fig. 4e) and has previously been shown to be crucial for functional integrity of the binding pocket^42^. Mutations in this position could possibly be caused by an accompanying affinity maturation for the fluorescent ligand used for selection. Interestingly, mutations in position H173 and R178 both occur linked together, yet never in combination with a mutation in position D153, which otherwise occurs frequently, possibly indicating that they together would be unfavourable. H173 is located at the end of TM helix 4, and R178 is located in the β-hairpin formed by extracellular loop 2 of the receptor. All three positions (H173, R178 and I204) were not mutated at a high frequency in SaBRE, indicating that the positions might be crucial especially for increased production in *E. coli*.

While these observed mutational frequencies indicate how frequent the most popular non-wt amino acid is at a given position of the OTR, the data do not hold any information on the degeneracy of non-wt amino acids at that position (i.e., they do not describe whether or not there are multiple new residue types observed). This metric is captured by the Shannon entropy, which can be calculated from the sequencing data. We calculated the Shannon entropy for each amino acid position along the OTR gene (Fig. 4a–d). To provide context for the less-than-intuitive entropy values, we additionally plotted hypothetical curves for the Shannon entropy assuming a uniform distribution for a given number of non-native amino acids. This formula (Eq. (1)) depends only on the frequency of the wt amino acid (f_wt_) in the respective position (i) and the total number (n) of non-wt amino acids observed.

Adapted Shannon entropy (S):

1 $$S = - f_{wt} \times {\text{ln}}\left( {f_{wt} } \right) - \mathop \sum \limits_{1}^{n} \frac{{1 - f_{wt} }}{n} \times {\text{ln}}\left( {\frac{{1 - f_{wt} }}{n}} \right).$$

By comparing the calculated values to the theoretical curves, we can determine, at a glance, roughly how many different mutations are observed at each position. For most mutated positions only a single non-wt amino acid is observed (because of the low mutation rate) and thus the observed Shannon entropies fall along the theoretical line for n = 1 (Fig. 4). This outcome is expected, based on the underlying statistical nature of library creation by epPCR resulting in few mutations, and the accompanying limitation posed by codon bias, limiting the type of codon changes.

Nonetheless, exceptions were observed. For example, one of the few exceptions with a high entropy, i.e., a position with multiple different amino acids occurring, was position D153 (Fig. 4). Position D153 was found be mutated to either tyrosine, asparagine, histidine, valine and even glycine. Therefore, in position D153 five out of the seven amino acids which are accessible with just one base pair change to the respective codon are observed. This high entropy emphasizes the detrimental effect the aspartate in position D153 apparently exerts on receptor integrity, as it seems that any other amino acid is preferred at this position.

To capture the diversity of each pool, we calculated the cumulative density function of cluster size (Fig. 3e). Cluster size here is defined as the number of observations of each unique sequence. Unsurprisingly, we observed a negative correlation between cluster size and frequency of occurrence, i.e., there are many observations of unique single sequences and only few observations of clusters of many identical sequences (Fig. 3e). For all NGS pools, there is a single, dominant cluster with ~ 100–1000 observations of one sequence, clearly indicating enrichment by the selection methods.

The SaBRE 2.4 pool is more enriched than the SaBRE 2.3 pool, as demonstrated by a higher fraction of large clusters and simultaneously a drop in the frequency of small clusters (Fig. 3e). Interestingly, after one sorting round with CHESS, the enriched pool *E. coli* 3.5 appears to be *more* diverse than its parental pool *E. coli* 3.4. This might be an effect caused by the new selection pressure applied during sorting for stability with CHESS and the subsequent need to amplify the receptor gene by PCR (introducing more mutations), as re-cultivation of course is impossible from the permeabilised bacterial cells.

**Figure 5:**
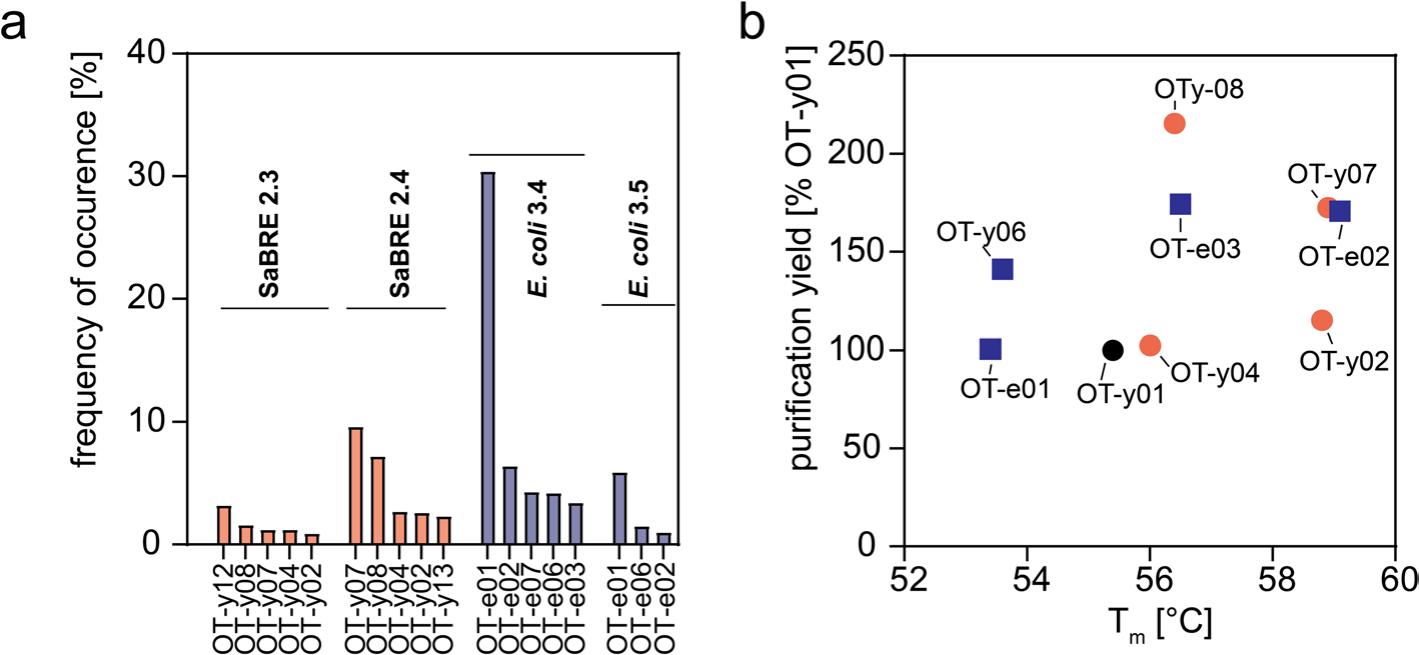
Characterization of most enriched single clones. (**a**) Occurrence of the most enriched variants of all four pools sequenced as determined by NGS. Variants identified in SaBRE pools (OT-yXX) are coloured in salmon, and variants identified in pools enriched during *E. coli*-based selection (OT-eXX) are coloured blue. Sequences were numbered according to their occurrence in initial Sanger sequencing results. (**b**) The purification yield of OTR purified from HEK293 cells, normalised to OT-y01, is compared to the apparent T_m_ derived from CPM-based thermostability measurements.

To finally assess purification yield and stability of the most enriched single OTR variants, they were expressed in HEK293T cells, and subsequently solubilised and purified. We probed purification yield and assessed thermostability by monitoring protein unfolding as a function of temperature using the previously described 7-diethylamino-3-(4-maleimidophenyl)-4-methylcoumarin (CPM) assay^43^ (Fig. 5, Supplementary Table S3). Because wtOTR could not be purified in sufficient yield, we could not determine its melting temperature (T_m_). Therefore, we selected the parental variant OT-y01 as reference for all tested OTR clones. As described previously, we found a trend that higher purification yield correlates with higher stability, even though this correlation is not very strong. Most tested variants show higher thermostability and expression yield compared to OT-y01, which indicates a clear selection for these properties.

Interestingly, we also observed that purification yields from mammalian cells does not seem to correlate with enrichment after SaBRE or selection in *E. coli*. The most stable clone tested was OT-e02 with a melting temperature of 59.2 °C and OT-y02 with a melting temperature of 58.8 °C. Noteworthy, OT-y02 is also the clone that served as the basis for the construct later successfully crystallised in LCP^42^.

## Discussion

GPCRs commonly require protein engineering to enable detailed biophysical studies and structure determination. While several receptors were sufficiently stabilized by using a particular directed evolution strategy, the most challenging receptors could not. The otherwise intractable human OTR serves as an example.

Among the available engineering strategies, CHESS, the selection method for stability in detergent, has proven to be an especially powerful technique for the identification of suitable receptor mutants. While mutants, stabilized through mutagenesis, can display conformational restraints compared to wt receptors^14^, their great potential is underlined by CHESS-improved GPCR mutants utilised in challenging biophysical and ligand-binding studies, including SPR^18,19^, NMR^44–46^ and X-ray crystallography^47^. However, until now, the limiting factor for applying CHESS has been that it requires some level of functional protein expression in *E. coli*.

Here we presented a strategy to make the otherwise intractable human OTR accessible to CHESS by a strategic combination of two directed evolution approaches: starting with selection for improved functional expression in yeast (SaBRE), followed by a subsequent selection for functional expression in *E. coli*, followed by CHESS. After transferring the SaBRE-selected clones to *E. coli*, we observed that the variants were still not expressing well in *E. coli*, precluding the direct application of CHESS.

In contrast, higher functional expression was retained when switching in the opposite direction, bringing mutants selected in *E. coli* or *S.* *cerevisiae* to a higher eukaryotic host, such as insect cells or mammalian cells^21,24^. A possible explanation for this observation might be the difference of protein quality-control machinery. Thus, a protein that is only able to fold in the presence of a complex machinery in higher eukaryotic cells would not fold in cells lacking that machinery, while the converse is not true, and a biophysically improved mutant may still show improvements in the presence of a more complex machinery.

With the goal of ultimately applying CHESS, which takes place in *E. coli*, the yeast-evolved variants provided an excellent starting point for subsequent selection for high expression in *E. coli,* while the wtOTR did not. We are confident that this strategy can be further adapted not only for other GPCRs, but also other membrane proteins that are initially toxic to *E. coli*. Such a stepwise strategy may be needed when membrane proteins need to be expressible in *E. coli*, e.g., for isotope labelling, or when they are to be examined in experiments which require long-time stability in a given detergent.

As we sought to determine the sequence features that led to enhanced stability in our evolution experiments, we turned to SMRT sequencing, an NGS approach that allowed us to sequence the entire 1056 bp target gene, and thus without losing the connections between mutations distant in the sequence. For this purpose, we developed a sequencing pipeline for the analysis of SMRT-sequencing-derived NGS libraries with very high accuracy, such that the very few true point mutations were clearly distinguishable from technical sequencing errors. With this approach we were able to acquire over 55,000 unique high-accuracy sequences from four different pools. By comparing sequences obtained from NGS with sequences obtained by Sanger sequencing, we were able to validate their correctness.

The limiting factor for the overall number of sequences obtained by our NGS approach was the low subread sequence quality, caused by the high occurrence of indels from SMRT sequencing. Consequently, we found the key to correctly assembling circular consensus sequencing reads was to first align each subread to the wt gene before generating the consensus sequence. Multiple strategies, programs, and parameters were empirically screened to find the best working solution. We restricted our approach to the use of pre-existing software, as the challenge of correctly identifying variants from indel-heavy data is an area of active research^48^ and beyond the scope of this manuscript.

To our knowledge, this is the first report of a long-read sequencing technology implemented to analyse DNA pools from directed evolution, where every single DNA molecule contains individual information. In future experiments, our NGS approach could simplify and accelerate the analysis of any diversified DNA pool. With the ability to simultaneously generate and analyse high sequence numbers, very quantitative and complete analyses of diverse pools become possible. Furthermore, this sequencing approach could provide a suitable platform to fully exploit selections based on rationally designed synthetic libraries, e.g., by removing the need for DNA barcodes for sequence identification in deep mutational scanning studies^49^.

Our analyses of the NGS-derived sequences allowed us to identify several co-enriched positions during the distinct evolution rounds. We observed D153X (where X = N, Y, V, G, & H, ordered according their observed frequency) and T333M to be present in several individual OTR variants enriched in yeast. Because of their high frequency of appearance, we reasoned that these mutations are particularly critical to the *S. cerevisiae*-based selection outcome. Conversely, in the sequences of *E. coli*-based OTR variants, we frequently observed either the co-occurrence of mutations H173Y and R178C or the mutation I204N. Interestingly, none of these three mutations are facing towards the helix bundle. Simultaneously, none of these three mutations were frequently observed in yeast-derived OTR variants. We thus speculate that these three mutations contribute to increased functional expression of the receptor in the *E. coli*, even though we cannot pinpoint yet the exact step where the optimized OTR performs better in the more demanding expression environment in *E. coli*.

With few exceptions, we found that most positions had only mutated to one other type of amino acid. This is most likely due to a fundamental limitation in exploring sequence space by epPCR, which will typically explore only explore codon changes accessible by a one-base mutation. As previously reported by Schlinkmann et al.^26,50^, comprehensive synthetic libraries containing all desired codons at given positions are needed to optimise the directed evolution outcome. With key positions identified in our selections and the subsequent elucidation of the OTR structure^42^, the rationalization of such binary libraries for the OTR as well as the closely related vasopressin receptors^51^ can now be successfully implemented. The transferability of these critical mutations from the OTR to vasopressin receptors could be directly assessed by grafting the corresponding mutations into the vasopressin receptors, similarly to the previously reported successful transfer of the SaBRE-derived mutations from the κ-opioid receptor^24^ to the δ-opioid receptor^52^.

In conclusion, while GPCRs with especially poor properties cannot be successfully optimised even with individual directed evolution technologies, we could show that an engineering strategy, based on the subsequent use of functional expression in yeast (SaBRE), followed by *E. coli*, and then the use of the especially powerful CHESS was able to solve this problem. The successful selection for improved and crystallisable variants of the human oxytocin receptor, a GPCR with particularly low intrinsic production levels, serves as an example. Our study has also shown, by the different nature of enriched mutations, that the evolution pressure on the same membrane protein in prokaryotes and eukaryotes is different. The very accurate long-read NGS strategy that we have developed for this purpose provides a general methodology for the highly accurate analysis of libraries of point mutants during directed evolution.

## Methods

### DNA library construction and transformation

Human OTR (2–389) was randomised using the GeneMorph II random mutagenesis kit (Agilent), aiming for a mean of two amino acid changes per gene. Two reactions with 200 ng template DNA were either amplified for 20 or 25 cycles, respectively. The created libraries from both reactions were column-purified (QIAquick PCR purification kit, Qiagen) and combined at a 1:1 molar ratio.

*S. cerevisiae* strain BY4741^53^ (EUROSCARF) was transformed as previously described^24^. In brief, sites homologous to the linearised yeast expression vector pMS003het^24^ (*Nhe*I/*Bam*HI digested) were added at each end of the OTR gene during epPCR by the primers, to allow homologous recombination in yeast. To obtain sufficient DNA yields for transformation, the created OTR libraries were further amplified by standard PCR. BY4741 cells were transformed by square wave electroporation on a GenePulser Xcell electroporator (BioRad) analogously to a previously published method^54^. After electroporation, cells were allowed to recover in YPD medium without shaking at 30 °C before pelleting and resuspending in 500 ml SDD-Leu-medium (Synthetic Defined Medium without Leu; 6.9 g L^−1^ yeast nitrogen base without amino acids (Formedium), 690 mg L^−1^ complete supplement mixture without leucine (Formedium), 20 g L^−1^ glucose, 35 mM sodium citrate tribasic, 35 mM citric acid) for selective growth at 30 °C for 24 h, and finally stored as glycerol stocks at − 80 °C until use. Libraries had a diversity of 5 × 10^7^ (SaBRE 1.0) and 7 × 10^7^ (SaBRE 2.0).

For efficient library creation in *E. coli*, restriction sites (*Bam*HI and *Spe*I) were introduced by the primers during amplification to both ends of the created library. To obtain the plasmid pEC01, pRG/III^55^ was modified by replacing the C-terminal thioredoxin and the polyhistidine-tag by an SGSGGGSG linker, followed by superfolder Green Fluorescent Protein (sfGFP)^56^ followed by a C-terminal Avi-tag. The amplified and digested library (*Bam*HI and *Spe*I) was ligated into linearised pEC01 overnight at 16 °C. Ligated vector was purified (QIAquick PCR purification kit, Qiagen) and eluted in distilled H_2_O. Electrocompetent DH5α cells (Thermo Fisher) were transformed with clean and concentrated ligation product by electroporation. Directly after electroporation, cells were allowed to recover in 4 mL SOC medium under shaking at 37 °C for 1 h. Thereafter, ampicillin (100 ng uL^-1^) was added, and cells were allowed to grow for another 2 h. To remove non-transformed cells, which fail to divide under ampicillin selection and grow to aggregating filaments instead, the recovered cells were passed through a 5-μm filter (Millipore). Cells collected from the flow-through were further incubated in 40 mL fresh 2YT containing 7% sucrose (w/v), 1% glucose (w/v) and 100 ng uL^−1^ ampicillin for 17 h and finally stored as glycerol stocks at − 80 °C. The total library size (*E. coli* 3.0) was 4 × 10^6^ as estimated from determining colonies in dilution series.

### Fluorescent ligand binding with yeast cells

A pre-culture was inoculated to OD_600_ = 0.2 in Synthetic Defined Medium without Leu (SDD-Leu^–^) and cultivated for 16–18 h at 30 °C. For expression, yeast cells from the pre-culture were collected and resuspended in SDG-Leu^–^ medium (identical to SDD-LEU^–^ with galactose instead of glucose) to an OD_600_ = 1.0 and incubated for 24 h at 20 °C. After expression, 5 × 10^8^ cells were collected and washed once in TELi (50 mM Tris–HCl pH 9.0 (at 4 °C), 100 mM lithium acetate, 1 mM EDTA), before resuspending in TELi supplemented with 50 mM DTT. To allow efficient permeabilization of the cell wall, cells were incubated at 20 °C for 30 min and finally washed twice in ice cold TELi. 1 × 10^8^ washed and permeabilised yeast cells were collected and resuspended in 2 mL TELi supplemented with 50 nM HiLyte Fluor 647-Lys^8^ PVA (Eurogentec) for 2 h on ice without exposure to light. To assess unspecific binding, corresponding samples were additionally supplemented with 2.5 μM unlabelled PVA as competitor. Before measuring flow cytometry, cells were collected by centrifugation and resuspended in 1 mL TELi.

### Fluorescent ligand binding with bacterial cells

A pre-culture was inoculated to an OD_600_ = 0.05 in LB medium containing ampicillin (100 ng μL^−1^) and 1% glucose (w/v) and cultivated for 2–3 h at 37 °C. The pre-culture was subsequently used to inoculate 5 mL 2YT containing 0.2% glucose and 100 ng μL^-1^ ampicillin to an OD_600_ = 0.05. When the expression culture had grown to OD_600_ = 0.5–0.6, expression was induced by addition of 250 μM IPTG and was allowed to proceed for 20 h at 20 °C. Then, 10^9^ cells were collected and washed twice with 1 mL ice-cold TKCl (50 mM Tris–HCl, 150 mM KCl, pH 7.4). 10^7^ washed cells were incubated in 250 μL TKCl containing 100 nM HiLyte Fluor 647-Lys^8^-PVA for 2 h on ice without exposure to light. To assess unspecific binding, corresponding samples were additionally supplemented with 2.5 μM unlabelled PVA for competition. Before measuring flow cytometry, cells were collected by centrifugation and resuspended in 200 μL TKCl.

### Fluorescent ligand binding with polymer-encapsulated solubilised bacterial cells

1 × 10^10^ receptor-expressing *E. coli* cells were collected and encapsulated with one layer of chitosan and one layer of alginate, strictly following the procedure published previously^22^. Encapsulated cells were solubilised in PBS-ED (137 mM NaCl, 2.7 mM KCl, 8.1 mM Na_2_HPO_4_, 1.8 mM KH_2_PO_4_, pH 7.4, 1 mM EDTA, cOmplete protease inhibitor EDTA-free tablets (Roche), 1% *n*-dodecyl-*β*-d-maltopyranoside (DDM, Anatrace) (w/v), 0.1% cholesteryl hemisuccinate (CHS, Sigma Aldrich) (w/v)) containing 50 nM HiLyte Fluor 647-Lys^8^-PVA (binding buffer) or 50 nM HiLyte Fluor 647-Lys^8^-PVA and 2.5 μM unlabelled PVA (competition buffer) at 4 °C for 1 h. Solubilised cells, i.e., polymer capsules, were washed once with either binding or competition buffer. Washed capsules were incubated for another 3 h in the respective buffer. Before carrying out flow cytometry measurements, capsules were collected by centrifugation and resuspended in 500 μL PBS-ED.

### Flow cytometry and FACS

Flow cytometry was performed on a BD FACSCanto II cytometer (BD Biosciences) and fluorescence-activated cell sorting was performed on a BD FACSAria III sorter (BD Biosciences). During FACS, the top 0.5% of the fluorescent events were retained. Cells were directly sorted into growth medium and subsequently cultivated to stationary phase at 30 °C (yeast) or 37 °C (bacteria), respectively. Capsules were sorted into distilled H_2_O and the DNA encoding OTR was amplified by PCR. Amplified DNA was re-cloned as described above. Flow cytometry data were analysed with FCS Express 5 Flow (De Novo Software).

### Thermostability measurements

HEK293T/17 cells (ATCC) were cultivated in Dulbecco’s modified medium (Sigma Aldrich) supplemented with 100 units/mL penicillin, 100 µg/mL streptomycin (Sigma Aldrich) and 10% (v/v) fetal calf serum (BioConcept). Cells were maintained at 37 °C in a humidified atmosphere of 5% CO_2_, 95% air. Transient transfections of OTR containing an N-terminal fused FLAG-tag were performed with TransIT-293 (Mirus Bio) according to the manufacturer’s protocol.

HEK293T cells were seeded and transfected in 6-well plates (Falcon) at a cell density of 1.5 × 10^6^ cells per well. 48 h after transfection cells were harvested, washed once in PBS (pH 7.4) and stored at − 20 °C. Purification of expressed OTR constructs was performed as published in detail (Schöppe et al., manuscript submitted). In brief, cells were lysed in hypotonic solution by resuspending in low salt buffer (10 mM HEPES pH 7.5, 20 mM KCl, 10 mM MgCl_2_, 50 µg mL^−1^ Pefabloc SC (Carl Roth), 1 µg mL^−1^ Pepstatin A (Carl Roth)) and subsequently solubilised in solubilization buffer (low salt buffer supplemented with 500 mM NaCl, 0.5% DDM (w/v), 0.1% CHS (w/v), 200 μM PVA). Insoluble cell debris was removed by centrifugation and the cleared supernatant was incubated with Anti-FLAG M2 magnetic beads (Sigma Aldrich) at 4 °C for 2.5 h. The receptor-bound resin was washed with 25 column volumes (CV) of Wash Buffer I (50 mM HEPES pH 7.5, 500 mM NaCl, 10 mM MgCl_2_, 10% (v/v) glycerol, 1.0% (w/v) DDM, 0.2% (w/v) CHS, 8 mM ATP, 50 µM PVA) followed by 35 CV of Wash Buffer II (50 mM HEPES pH 7.5, 500 mM NaCl, 10% (v/v) glycerol, 0.05% (w/v) DDM, 0.01% (w/v) CHS, 50 µM PVA). PVA-bound OTR was eluted in one CV of Elution Buffer (50 mM HEPES pH 7.5, 500 mM NaCl, 3 mg mL^−1^ 3× FLAG Peptide (Sigma Aldrich), 10% (v/v) glycerol, 0.05% (w/v) DDM, 0.01% (w/v) CHS, 50 µM PVA). CPM dye was added to eluted pure PVA-bound OTR and thermostability was assessed on a Mx3005P QPCR System (Agilent) qPCR machine. Data were analysed GraphPad Prism software (version 8.1.1, GraphPad Prism).

### SMRT sequencing

The OTR genes (2–353) enriched in pools SaBRE 2.3, SaBRE 2.4, *E. coli* 3.4 and *E. coli* 3.5 were amplified by PCR and individualised and simultaneously circularised by ligating a unique barcode adapter using the SMRTbell Express Template Prep Kit 2.0 (PacBio) and Barcoded Overhang Adapters (PacBio) (Supplementary Fig. S2b). Amino acids 354–389 of the OTR C-terminus were excluded from analysis to increase overall CCS read accuracy by shortening the subread length and thus consequently increasing the mean number of subreads achieved from a single read. Sequencing was performed on a PacBio Sequel instrument (Pacific Bioinformatics) using one 1 M SMRT cell (Pacific Bioinformatics) as service by the Functional Genomics Centre Zurich (FGCZ).

## Supplementary Information

Supplementary Information.
