# Supplementary material for: Directed evolution for high functional production and stability of a challenging G protein-coupled receptor
Source: Sci Rep. 2021 Apr 21;11:8630. doi: 10.1038/s41598-021-87793-9 (PMC8060309; doi:10.1038/s41598-021-87793-9)
Supplement: Supplementary file 1 — Supplementary Information. [file 41598_2021_87793_MOESM1_ESM.pdf]

Supplementary Material

Directed evolution for high functional production and stability of a challenging G protein-coupled receptor

Yann Waltenspühl[1], Jeliazko R. Jeliazkov[1], Lutz Kummer[1], and Andreas Plückthun[1].

[1] *Department of Biochemistry, University of Zürich, Winterthurerstrasse 190, CH-8057 Zürich, Switzerland.*

Correspondence and requests for materials should be addressed to A.P. (plueckthun@bioc.uzh.ch)

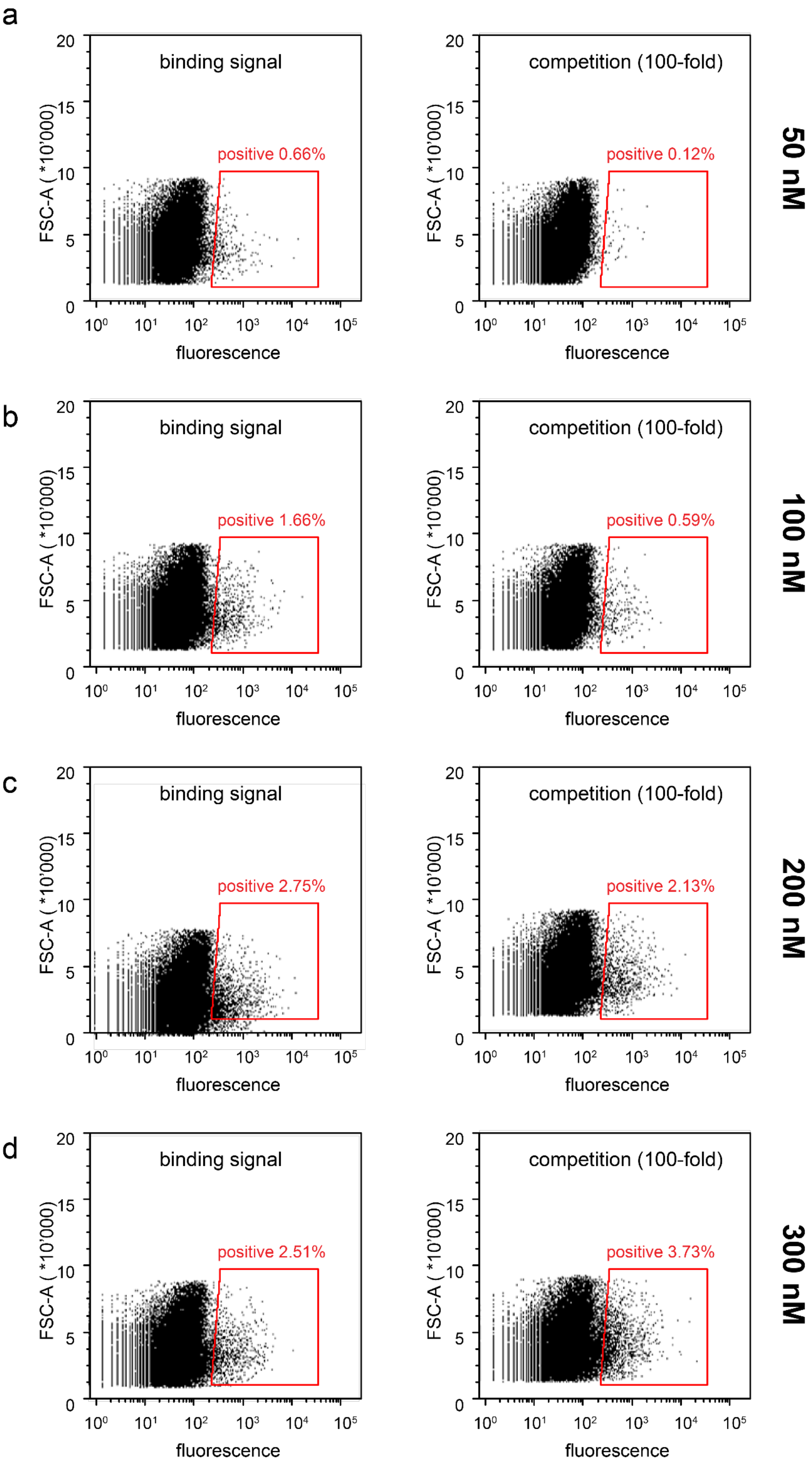
a
b
c
d
binding signal
competition (100-fold)
positive 0.66%
positive 0.12%
positive 1.66%
positive 0.59%
positive 2.75%
positive 2.13%
positive 2.51%
positive 3.73%
FSC-A (*10'000)
fluorescence
50 nM
100 nM
200 nM
300 nM

Supplementary Figure S1. Optimizing fluorescent ligand probe concentration in flow cytometry. (**a - d**) Flow cytometry data of fluorescent ligand binding to the GPCR, with total binding signal (left panels) and nonspecific signal (competition with unlabelled ligand, right panels) are shown for *S. cerevisiae* expression pool SaBRE 1.0. Cells are probed with increasing concentrations of fluorescent ligand to identify optimal ligand concentration for sorting.

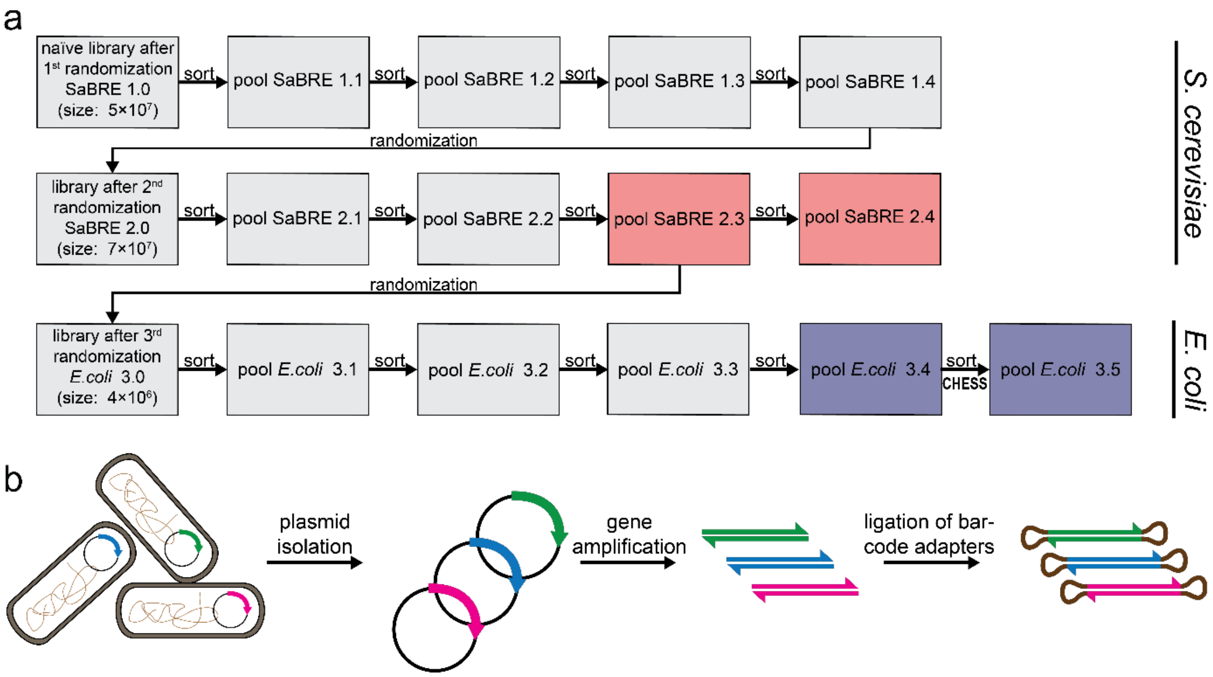

Supplementary Figure S2. Overview OTR selection procedure. (**a**) Overview of the selection process. Each square indicates a unique DNA pool, a right-pointing arrow indicates a selection step conducted with FACS ("sort"), a left-pointing arrow indicates a randomization by epPCR. DNA pools subjected to analysis by sequencing are coloured in salmon (SaBRE) or purple (*E. coli*-based evolution for functional expression or CHESS). (**b**) Overview of OTR DNA (coloured arrows) preparation to allow analysis with NGS. These steps consist of plasmid isolation, amplification of OTR (2-353) by PCR, followed by circularization of amplified variants by ligation of individual barcode adapters (brown circles).

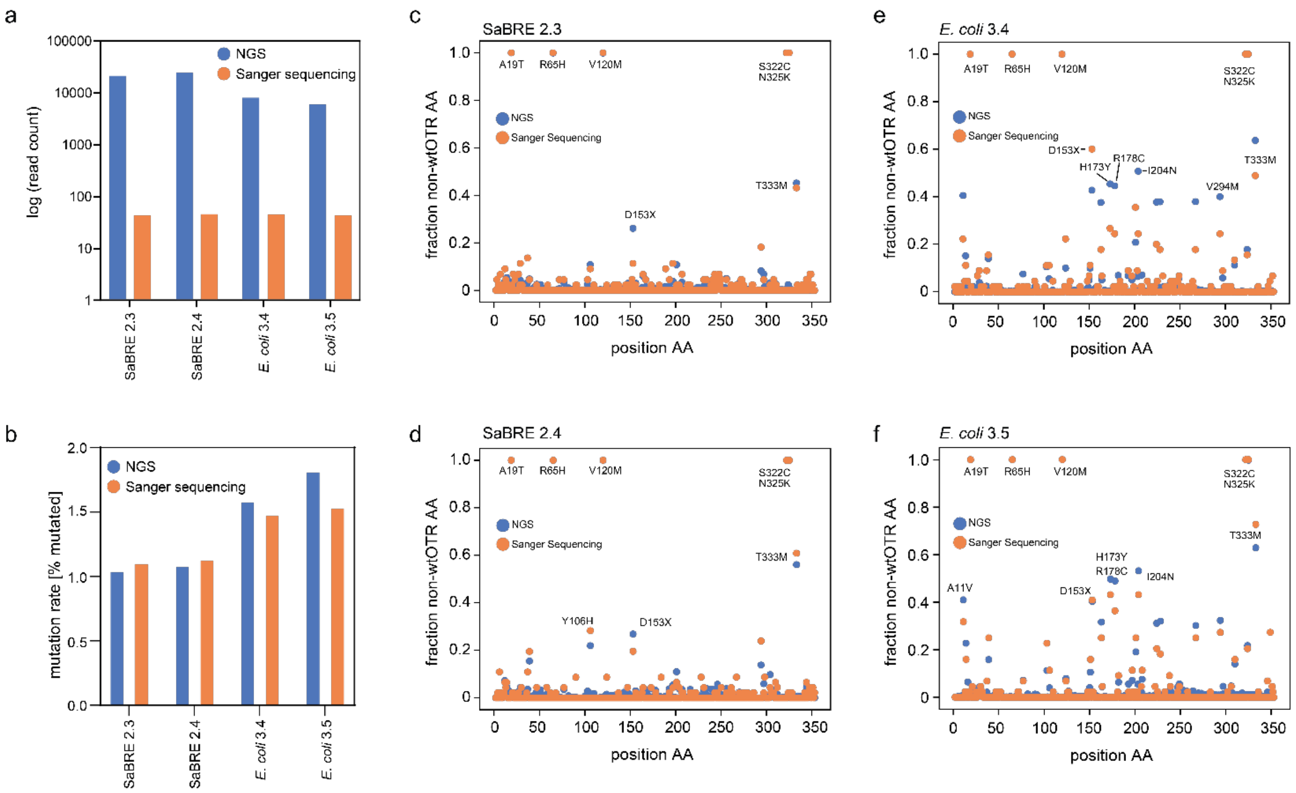

Supplementary Figure S3. Comparison of sequence data obtained from NGS and Sanger sequencing. (**a**) Comparison of total sequence reads obtained by NGS (blue bars) or Sanger sequencing (orange bars) for all four pools sequenced. (**b**) Comparison of the average base pair mutation rate frequency calculated for sequences obtained by NGS (blue bars) or Sanger sequencing (orange bars) for all four pools sequenced. (**c – f**) Frequency of non-wt OTR amino acid (AA) present in every position along the OTR as calculated for sequences obtained by NGS (blue bars) or Sanger sequencing (orange bars) for (c) SaBRE 2.3, (d) SaBRE 2.4, (e) *E. coli* 3.4 or (f) *E. coli* 3.5.

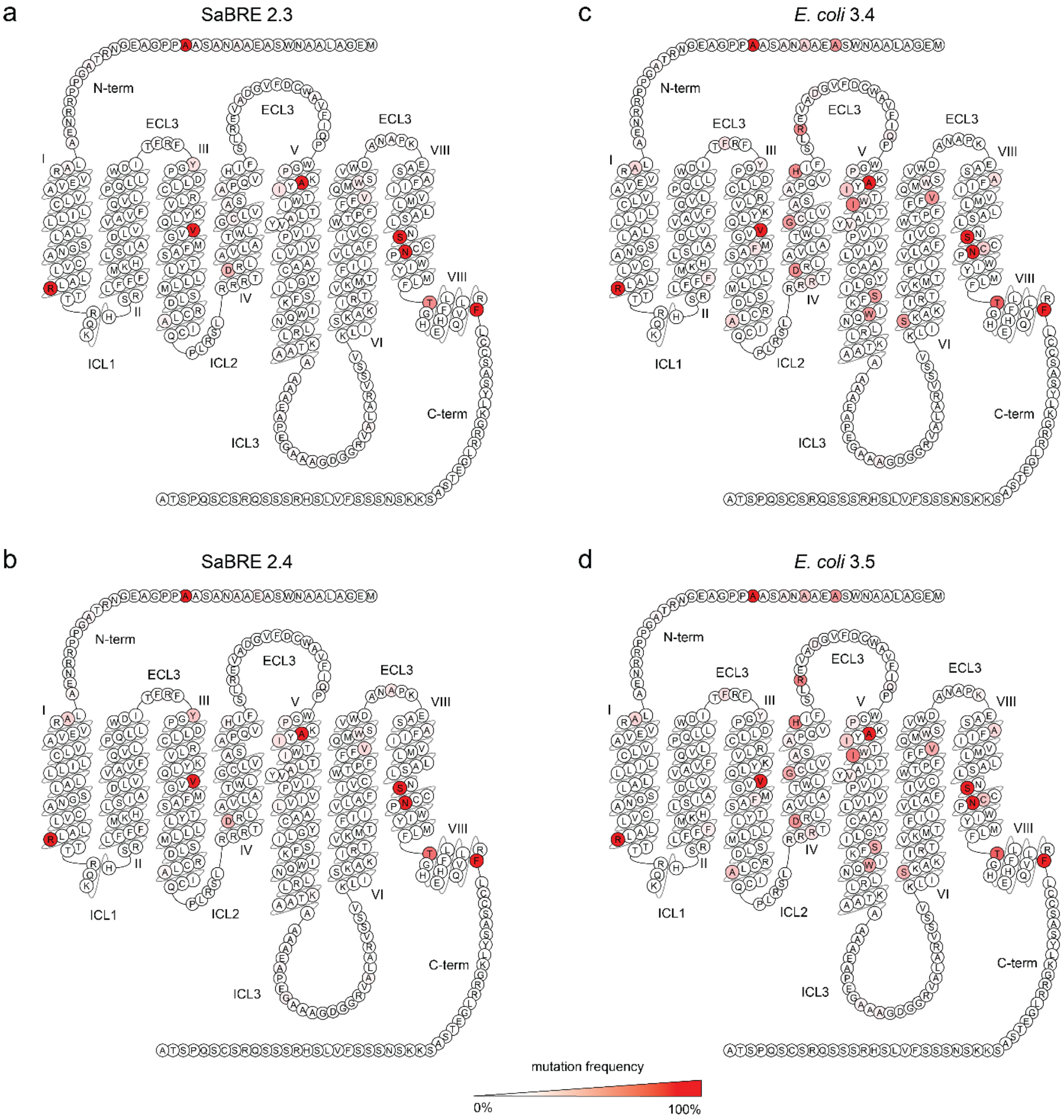

Supplementary Figure S4. Sequence variability in analysed pools. (**a-d**) Positional sequence variability is coloured from low (white) to high mutation frequency (red) in snake plots as observed in pool (a) SaBRE 2.3, (b) SaBRE 2.4, (c) *E. coli* 3.4 or (d) *E. coli* 3.5.

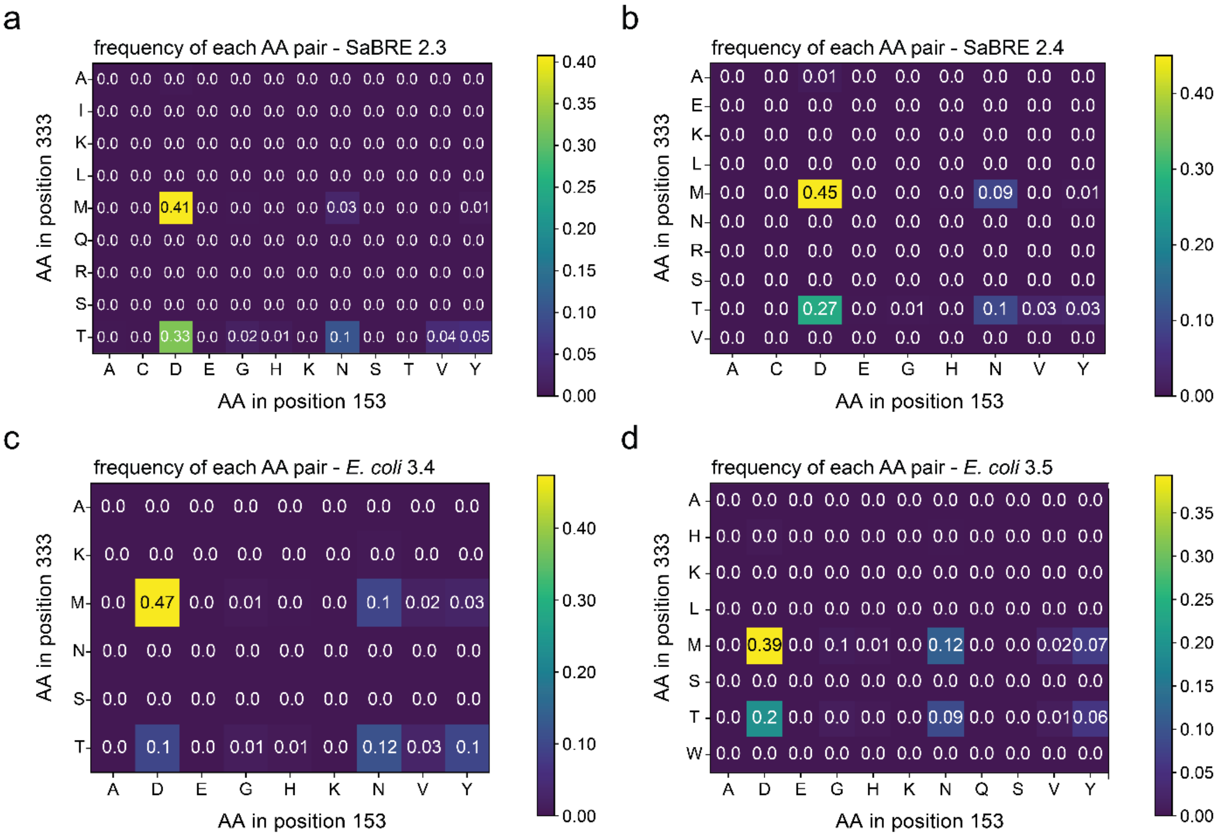

Supplementary Figure S5. Observed pair frequencies. (**a-d**) Positional pair frequency of amino acid (AA) positions 153 and 333 is coloured from low (purple) to high frequency (yellow). Paired plots were calculated for individual pools (a) SaBRE 2.3, (b) SaBRE 2.4, (c) *E. coli* 3.4 or (d) *E. coli* 3.5.

Supplementary Table S1. Overview of the most enriched clones after selection

Ballesteros -Weinstein	N	N	N	N	1.32	1.58	2.42	EL1	3.19	3.33	3.37	4.39	4.40	4.42	4.52	4.56	4.62	EL2	EL2	EL2	5.39	5.41	5.42	5.46	5.54	5.62	5.66	5.72	IL3	IL3	IL3	6.27	6.54	EL3	7.32	7.34	7.46	7.48	7.49	8.47	8.58	
**variant**	**11**	**14**	**16**	**19**	**39**	**65**	**77**	**103**	**106**	**120**	**124**	**150**	**151**	**153**	**163**	**167**	**173**	**178**	**182**	**193**	**201**	**203**	**204**	**208**	**216**	**224**	**228**	**234**	**243**	**246**	**249**	**267**	**294**	**304**	**308**	**310**	**322**	**324**	**325**	**333**	**344**	**total mutations**
wtOTR	A	A	A	A	A	R	F	F	Y	V	F	R	R	D	G	A	H	R	D	Q	I	W	I	V	L	S	W	K	A	G	A	S	V	A	A	A	S	C	N	T	F	
OT-y01				T		H				M																											C		K			5
OT-y02				T		H				L						V				R								R	E	D							C		K	M		11
OT-y04				T		H				M																							M				C		K	M	V	8
OT-y07				T	V	H			H	M																											C		K	M		8
OT-y08				T		H				M				N																				E			C		K	M		8
OT-y12				T		H				M																											C		K	M		6
OT-y13				T		H				M							Y	C					N		H					D					D		C		K			11
OT-e01	V			M		H				M					V		Y	C					N			N	R					P	L				C		K	M		15
OT-e02				T	V	H		V		M			H	N							V															T	C	Y	K			12
OT-e03				T		H				M	Y	H		Y						K		C									V						C		K			11
OT-e06		I		T		H				M							Y	C					N														C		K	M		10
OT-e07		V		T		H	Y			M				N					N				N	L													C	Y	K	M		13

Mutations of each clone are indicated by the sequential amino acid numbering and Ballesteros-Weinstein (B.-W.) numbering.

Supplementary Table S2. SMRT sequencing data

pool	ZMW count	$n_{subreads}$ > 20	n_{reads} correct lenght	n_{reads} passed QC	NGS mutation rate (%)	Sanger mutation rate (%)
**OTR$_{SaBRE\ 2.3}$**	129,938	54,766	30,287	21,443	1.05	1.11
**OTR$_{SaBRE\ 2.4}$**	144,889	61,878	33,887	24,323	1.09	1.14
**OTR$_{\textit{E. coli}\ 3.4}$**	117,786	50,390	13,064	8,104	1.59	1.49
**OTR$_{\textit{E. coli}\ 3.5}$**	125,511	53,981	11,391	6,037	1.82	1.54

Summary of reads generated during SMRT sequencing (zero mode waveguide counts) of enriched DNA pools and the individual number (n) of sequences retained during or after individual data processing steps, respectively. Mutational rates of sequences generated by NGS or Sanger sequencing were determined by the number of mutated base pairs divided by the total numbers of base pairs within a pool

Supplementary Table S3. Overview most frequently occurring clones in the selection pools

pool	name	rank in Sanger	counts	frequency (%)	T_m [°C]-PVA	yield from HEK293T (% OTY01)
OTR_{SaBRE1}	OT-y01				55.4	100.0
$OTR_{SaBRE\ 2.3}$	OT-y12	2	679	3.2	n.d.	n.d
	OT-y08		332	1.6	56.4	215.4
	OT-y07		249	1.2	58.9	172.5
	OT-y04	1	241	1.2	56.0	102.4
	OT-y02	1	187	0.9	58.8	115.0
$OTR_{SaBRE\ 2.4}$	OT-y07	1	2211	9.6	58.9	172.5
	OT-y08	4	1677	7.2	56.4	215.4
	OT-y04		629	2.7	56.0	102.4
	OT-y02		591	2.6	58.8	115.0
	OT-y13		520	2.3	n.d.	n.d.
$OTR_{E.\ coli\ 3.4}$	OT-e01	1	2438	30.4	53.4	100.5
	OT-e02	2	516	6.4	59.1	170.7
	OT-e07		343	4.3	n.d.	n.d.
	OT-e06	4	336	4.2	53.6	141.0
	OT-e03	3	270	3.4	56.5	174.3
$OTR_{E.\ coli\ 3.5}$	OT-e01	2	350	5.9	53.4	100.5
	OT-e06	1	89	1.5	53.4	141.7
	OT-e02	1	60	1	59.4	133.3

Characterization of most enriched single clones within the four DNA pools analysed by NGS.
